# A serine/threonine phosphatase, PP2A, controls autoimmunity

**DOI:** 10.1186/ar4646

**Published:** 2014-09-18

**Authors:** George C Tsokos

**Affiliations:** 1 Harvard Medical School, Beth Israel Deaconess Medical Center, Boston, MA, USA

## 

Although tyrosine phosphatases have been well documented to be involved in the control of the immune system, serine/threonine phosphatases have not been assigned a similar role. Studies in patients with systemic lupus erythematosus (SLE), however, revealed that the protein, mRNA and catalytic activity of a serine/threonine phosphatase A (PP2A) are increased in T cells.

More importantly, PP2A has a master role in the aberrant biochemistry of SLE T cells because it: dephosphorylates pCREB and deprives an enhancer from various genes; dephosphorylates Elf1 and accounts for decreased expression of CD3ς and increased expression of FcRγ; activates (dephosphorylates) SP1 and promotes the expression of SP1-dependent genes such as CREM; and suppresses the activity of DNMT1 and promotes gene demethylation.

Gene function studies have revealed that promoter and intronic SNPs, along with epigenetic modifications of the promoter region, account for the expression of increased amounts of PP2A in SLE patients.

A mouse overexpressing PP2Ac in T cells does not develop autoimmunity but it displays increased amounts of IL-17 in the blood and develops florid glomerulonephritis when challenged with an anti-GBM antibody. Lastly, PP2A is involved in the epigenetic control of expression of IL-17.

Besides the increased expression of the catalytic subunit of PP2A, several regulatory (B) subunits appear to be abnormally expressed in SLE patients. Specifically, Bb' is decreased in one-half of SLE patients and accounts for defective IL-2 deprivation T-cell death, thus prolonging the survival of autoreactive T cells.

In conclusion, the catalytic and regulatory subunits of PP2A are abnormally expressed in SLE and contribute to aberrant T-cell function and organ damage. The fact that PP2Ac controls several downstream events urges its therapeutic targeting. The findings that PP2A B subunits control distinct T-cell function will enable corrective actions without the fear of collateral damage.

